# An Algae-Made RBD from SARS-CoV-2 Is Immunogenic in Mice

**DOI:** 10.3390/ph15101298

**Published:** 2022-10-21

**Authors:** Dania O. Govea-Alonso, Ashwini Malla, Omayra C. Bolaños-Martínez, Sornkanok Vimolmangkang, Sergio Rosales-Mendoza

**Affiliations:** 1 Laboratorio de Biofarmacéuticos Recombinantes, Facultad de Ciencias Químicas, Universidad Autónoma de San Luis Potosí, Av. Dr. Manuel Nava 6, San Luis Potosi 78210, Mexico; 2 Sección de Biotecnología, Centro de Investigación en Ciencias de la Salud y Biomedicina, Universidad Autónoma de San Luis Potosí, Av. Sierra Leona 550, Lomas 2ª Sección, San Luis Potosi 78210, Mexico; 3 Department of Pharmacognosy and Pharmaceutical Botany, Faculty of Pharmaceutical Sciences, Chulalongkorn University, Bangkok 10330, Thailand; 4 Center of Excellence in Plant-Produced Pharmaceuticals, Chulalongkorn University, Bangkok 10330, Thailand

**Keywords:** mucosal vaccine, *Chlamydomonas reinhardtii*, oral vaccine boosting, variant of concern, sterilizing immunity

## Abstract

Despite the current advances in global vaccination against SARS-CoV-2, boosting is still required to sustain immunity in the population, and the induction of sterilizing immunity remains as a pending goal. Low-cost oral immunogens could be used as the basis for the design of affordable and easy-to-administer booster vaccines. Algae stand as promising platforms to produce immunogens at low cost, and it is possible to use them as oral delivery carriers since they are edible (not requiring complex purification and formulation processes). Herein, a *Chlamydomonas*-made SARS-CoV-2 RBD was evaluated as an oral immunogen in mice to explore the feasibility of developing an oral algae-based vaccine. The test immunogen was stable in freeze-dried algae biomass and able to induce, by the oral route, systemic and mucosal humoral responses against the spike protein at a similar magnitude to those induced by injected antigen plus alum adjuvant. IgG subclass analysis revealed a Th2-bias response which lasted over 4 months after the last immunization. The induced antibodies showed a similar reactivity against either Delta or Omicron variants. This study represents a step forward in the development of oral vaccines that could accelerate massive immunization.

## 1. Introduction

The COVID-19 pandemic represents a dramatic issue for the global health, which has generated, as of August 2022, nearly 6.5 million deaths and more than 600 million reported cases [1]. This problem has been aggravated as variants of concern emerged. The accelerated efforts to develop vaccines and get their approval has led to the emergency use of several formulations that have been administered in intensive vaccination campaigns, setting a historic achievement for vaccinology, and the immunity achieved against SARS-CoV-2 in the global population stands as a milestone of modern vaccinology [2,3]. Approximately 322 vaccine candidates against COVID-19 are under investigation in various clinical or preclinical stages; from these candidate vaccines, only eleven have been approved for human use. Most of these vaccines are based on the Spike (S) protein or the RBD region (Receptor Binding Domain) of this protein, as targets, with the aim of inducing neutralizing antibodies and T cell responses that neutralize the infection; these approved vaccines are based on mRNA, inactivated viruses, proteins subunit, and adenoviral vectors [4,5,6]. To this date, COVID-vaccines used for this emergency health problem present some limitations: for example, they require boosting since humoral responses decay over time [7]; the cold-chain must never be interrupted, specifically for mRNA vaccines [6,8]; and low efficiency could exist for adenoviral vector-based vaccines due to the preexisting immunity to the vectors [9]. In addition, data on the efficacy and safety of these vaccines in people with comorbidities and preexisting health conditions, such as pregnancy or diabetes, are limited.

Despite these achievements, understanding the possible mechanisms leading to sterilizing immunity and developing thermostable vaccines to facilitate massive vaccination champaigns are pending objectives. Mucosal immunization has been proposed as an approach to induce robust humoral responses in the mucosal compartments that could block early virus spread and thus avoid the infection onset [10,11]. Therefore, intensive research on this direction is needed. Several groups are exploring mucosal vaccines based on adenoviral vectors, nanoparticles, live attenuated virus, and subunit vaccines, among other technologies [12,13,14,15,16,17,18,19,20,21].

Microalgae have been used over the last decades as promising platforms to produce biopharmaceuticals. Although no clinical trials have been conducted with algae-made vaccines, the advantage of the system justifies a deep exploration of this platform for emergent pathogens. Among the expression approaches, chloroplast and nuclear expression have been established through adopting several DNA transfer technologies, mainly biolistics, electroporation, and *Agrobacterium* infection [22,23,24]. Overall, stable transformation requires extensive characterization of the candidate clones to identify the most productive and stable ones, which is mainly due to the random insertion of the heterologous DNA into the genome. In contrast, the recently reported transient expression technologies offer rapid production and high yields by not requiring the characterization of clones (e.g., geminiviral vectors such as pAlgevir and BeYDV vectors) [25,26,27].

Our group previously reported the expression of RBD in two algae species (*Chlamydomonas reinhardtii* and *Chlorella vulgaris*), rendering algae-made RBD versions with proven antigenic activity [28]. In the present study, the immunogenicity of the algae-made RBD was assessed in test mice. Oral and injected formulations were characterized in terms of their ability to induce specific humoral responses against the S protein in both systemic and mucosal compartments. The IgG subclasses and humoral response were also analyzed.

## 2. Results

### 2.1. The Algae-Made RBD Is Highly Stable in the Freeze-Dried Biomass

**Figure 1:**
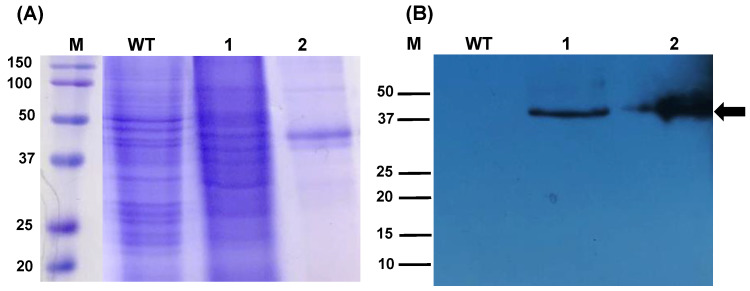
Stability of the RBD protein expressed transiently in *C. reinhardtii*. The total soluble protein (TSP) was extracted from dry biomass stored at room temperature for 15 months. (**A**) SDS-PAGE pattern of the TSP. Gel stained with Coomassie brilliant blue. (**B**) Western blot analysis labelled with anti-His tag antibody. Lanes; M: Molecular weight marker, WT: Wild-type, Lane 1: TSP from *C. reinhardtii* expressing RBD, Lane 2: RBD purified from *N. benthamiana* (6.5 µg) used as positive control.

The algae-made RBD used in this study was obtained following a previously reported transient expression technology, which is based on the Agrobacterium-mediated transfer of a geminiviral replicon. Algae cultures were infected with the agrobacteria strain carrying the RBD expression vector and the transformed algae biomass was screened for the presence of the target antigen [28]. The biomass was freeze-dried, and the obtained powder was stored for a long period of time (15 months) at room temperature. To validate the stability of the RBD antigen contained in the algae biomass, the total soluble protein obtained from the freeze-dried material was analyzed by Western blot to evaluate its integrity, revealing the presence of the 38 kDa immunoreactive protein with no smears that typically indicate protein degradation (Figure 1). This finding suggests that the RBD is highly stable in the algae cell matrix and justifies the performance of immunogenicity assessment.

### 2.2. Algae-Made RBD Induced Both Systemic and Mucosal Responses

**Figure 2:**
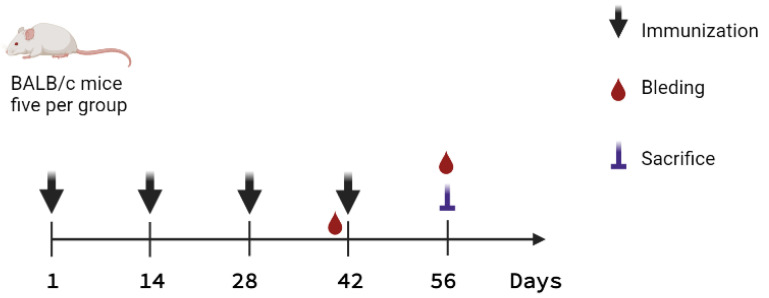
Immunization scheme to characterize the algae-made RBD. Mice were immunized by either oral or subcutaneous route on days 1, 14, 28, and 42 (denoted by the black arrows); and blood samples were withdrawn on days 41 and 56. Animals from PBS and C. RBD + CT p.o. groups were sacrificed until day 200 to determine long-lasting humoral responses.

**Figure 3:**
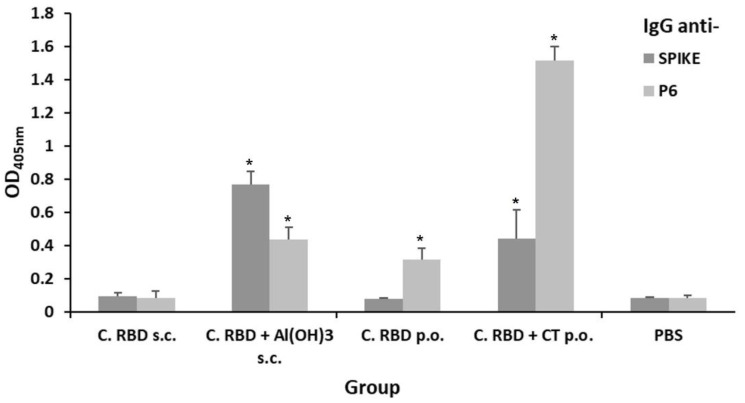
Evaluation of the systemic humoral response (total IgG) induced by the algae-made RBD. The IgG levels were measured in sera samples (1:100 dilution) by ELISA using either spike or P6 as the target antigen. Treatments for each experimental group are described in Table 1. Error bars represent standard deviations. * denotes statistically significant differences versus the PBS group (*p* < 0.05).

Mice were immunized by the subcutaneous route with total soluble protein extracts or by the oral route with suspensions of freeze-dried algae, alone or in presence of adequate adjuvants, following the immunization schedule presented in Figure 2 and Table 1. Humoral immune responses were evaluated on day 56. The s.c.-administered algae-made RBD induced very a low response when compared to the formulation supplemented with alum, in terms of IgG levels against either the S_461-493_ peptide (P6) or Spike protein. Interestingly, oral immunization with algae-made RBD plus Cholera toxin (CT) induced a higher anti-P6 IgG response when compared to the oral formulation lacking adjuvant and the subcutaneously immunized groups (Figure 3).

**Figure 4:**
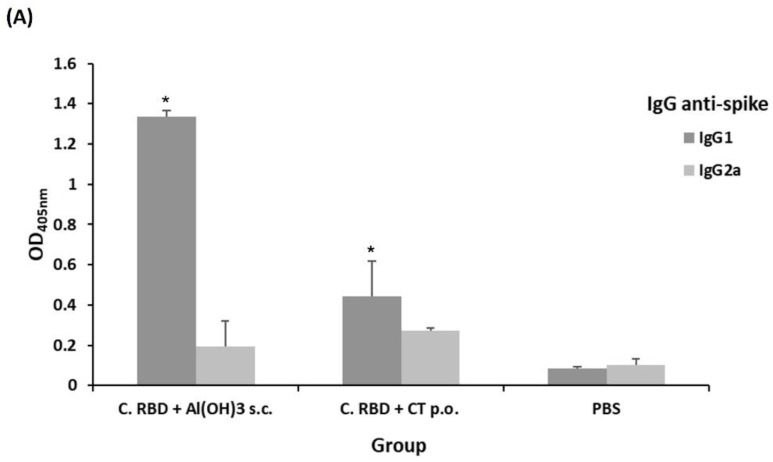

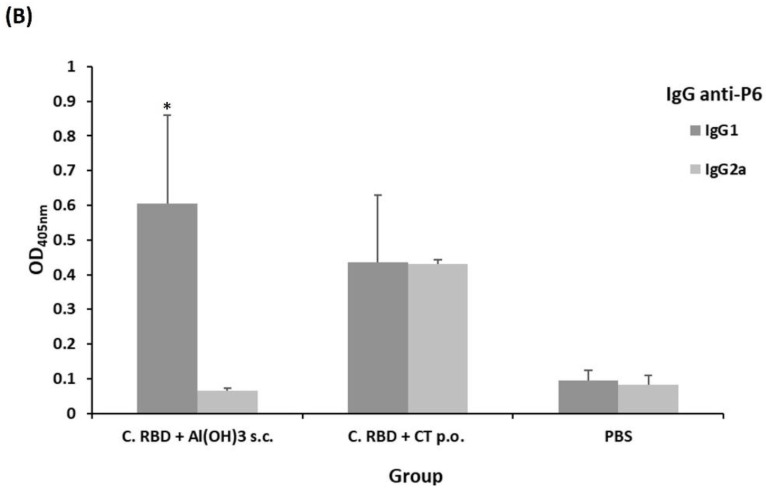
Characterization of the systemic humoral response induced by the algae-made RBD. The IgG subclasses, IgG1, and IgG2a were measured in serum samples (1:100 dilution) by ELISA using either spike (**A**) or P6 (**B**) as the target antigen. Error bars represent standard deviations. * denotes statistically significant differences versus the mean values of IgG2a measurements (*p* < 0.05).

The levels of IgG subclasses were evaluated in order to evidence the type of immune response that the algae-made RBD induces in mice. Interestingly, similarly high levels of anti-P6 IgG1 subclass were observed for C. RBD + CT p.o. and C. RBD + Al(OH)_3_ s.c. groups, whereas the PBS control group showed a very low signal (Figure 4B). When anti-spike antibodies were evaluated, higher levels of IgG1 subclass were observed in the C. RBD + Al(OH)_3_ s.c. group compared to the C. RBD + CT p.o. group (Figure 4A). Overall, these results suggest the induction of a Th2-bias immune response.

**Figure 5:**
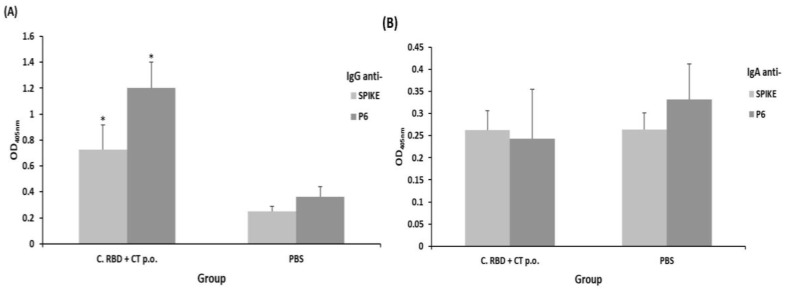
Evaluation of humoral response (IgA and IgG) at the mouth induced by the algae-made RBD. IgG (**A**) and IgA (**B**) antibodies were measured in saliva (1:4 dilution) by ELISA using either spike or P6 as the target antigen. Error bars represent standard deviations. * denotes statistically significant differences versus the PBS group (*p* < 0.05).

**Figure 6:**
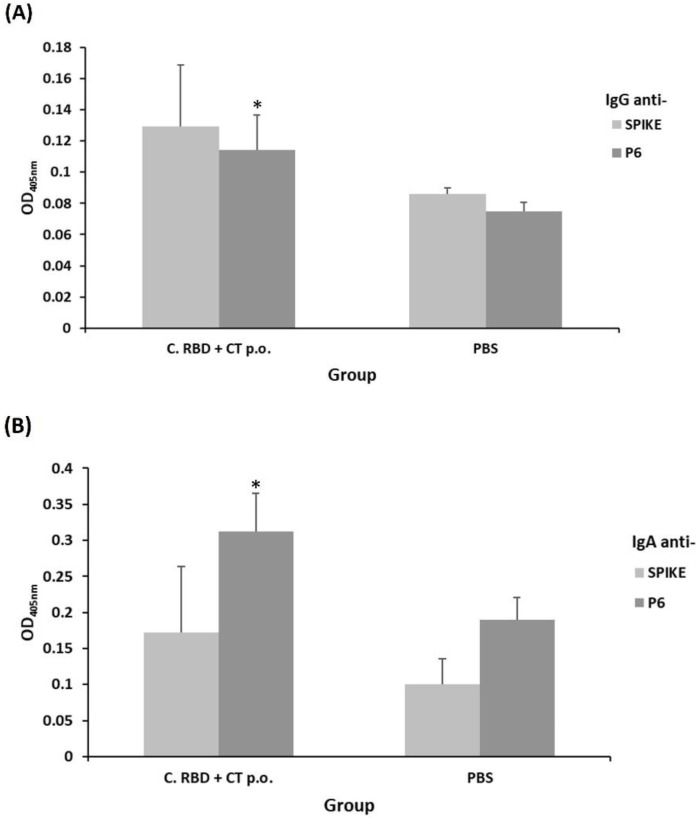
Evaluation of intestinal humoral response (IgA and IgG) induced by the algae-made RBD. IgG (**A**) and IgA (**B**) antibodies were measured in feces (1:2 dilution of the feces extract) by ELISA using either spike or P6 as the target antigen. Error bars represent standard deviations. * denotes statistically significant differences versus the PBS group (*p* < 0.05).

The IgG and IgA responses were evaluated at the mucosal level in feces and saliva samples in the PBS and C. RBD + CT p.o. groups. In saliva samples, the C. RBD + CT p.o. group showed a significant IgG response against both P6 and Spike protein (Delta) (Figure 5A). No statistical differences were observed for IgA antibodies in saliva samples (Figure 5B). Feces were also analyzed for measurement of IgA and IgG responses, and the induction of significant anti-P6 IgG levels was observed, whereas the PBS group showed a very low signal (Figure 6A). In addition, anti-P6 IgA response was observed in the C. RBD + CT p.o. group (Figure 6B). These results revealed the induction of relevant humoral immune response at both the systemic and mucosal levels.

### 2.3. Algae-Made RBD Induced a Long-Lasting Response of Antibodies Recognizing Delta and Omicron Spike Variants

**Figure 7:**
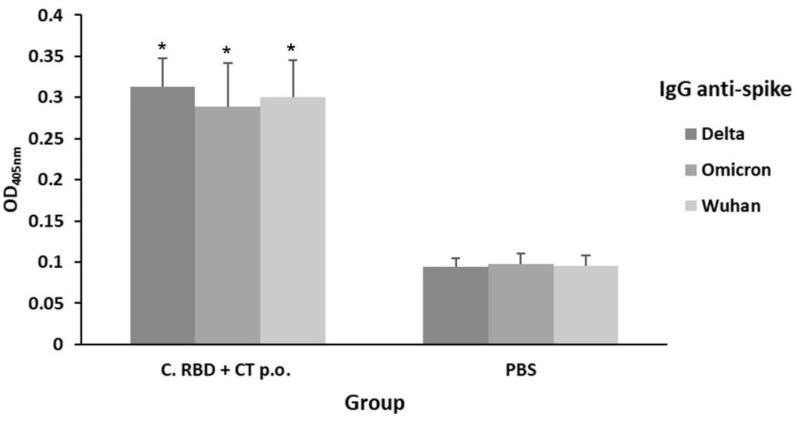
Evaluation of binding activity against SARS-CoV-2 variants and long-lasting response induced orally by the algae-made RBD. The IgG levels were measured in the C. RBD + CT p.o. test mice group on day 200. Sera samples (1:100 dilution) were analyzed by ELISA using either Wuhan, Delta, or Omicron Spike proteins as the target antigens. Error bars represent standard deviations. * denotes statistically significant differences versus the PBS group (*p* < 0.05).

In order to evaluate whether the algae-made RBD induces a long-lasting humoral response, mice from the PBS and C. RBD + CT p.o. groups were sampled on day 200, which is four months after the last boosting. ELISA revealed that significant anti-S (Delta variant) antibody levels are still detectable at this time point, suggesting a long-lasting immune response, whereas the PBS control group showed a very low background signal. In addition, ELISA-targeting the S protein from the relevant Omicron variant was also conducted, observing that the C. RBD + CT p.o. group developed a significant signal of similar magnitude to that observed for the Delta variant (Figure 7). A similar signal was observed for the Wuhan S protein. These results revealed that the algae-made RBD is capable of inducing antibodies with positive binding activity against Wuhan isolate and both Delta and Omicron SARS-CoV-2 variants, suggesting broad coverage against COVID-19.

## 3. Discussion

Herein the immunogenicity of the algae-made RBD was assessed in test mice as an effort to advance the development of functional vaccines against SARS-CoV-2. The performed immunization scheme aimed at comparing the humoral response induced by the test antigen co-administered with a conventional adjuvant (alum), which can be used without patent limitation, to that induced by oral immunization with CT as adjuvant. Oral immunization was explored as a convenient method in terms of easy administration and a pain-free approach that, in addition, does not require sterile devices for delivery. In general, when a soluble antigen is used, oral immunization is performed with a much higher antigen dose with respect to parenteral immunization. However, in our case, the oral formulation contains the target antigen encapsulated in the algal cells, which protect the antigen from degradation and eventually mediate its release if the digestion process occurs. This phenomenon could explain the fact that very low oral doses of the target antigen induce significant humoral responses as has been evidenced in other prototypes based on algae or plant cells administered by the oral route [29,30]. Therefore, we decided to use the same dose regardless of the immunization route.

Considering that the humoral response targeting the S protein is one of the correlates of protection for SARS-CoV-2 infection [31], we focused on determining the immunogenic potential of the algae-made RBD in terms of the induction of IgG responses against the target antigen, protein S. Interestingly, the injected algae-made RBD induced significant humoral response at the systemic level (serum), indicating that the antigen preserves the ability to induce antibodies able to bind the native target (spike protein). Since local mucosal immunity also plays a key role as it contributes to reducing the risk of infection and amelioration of disease severity; humoral responses in saliva were evaluated in mice orally immunized with the algae-made RBD, which not only induced a similar systemic IgG response compared to the injectable vaccine containing alum, but also induced significant IgG responses in saliva and IgG and IgA in feces, suggesting the induction of a relevant immune response at both levels. These data suggest that the algae-made RBD could be used for the design of oral vaccine formulations against SARS-CoV-2. Considering that CT is not an adjuvant approved for human use, further studies could contemplate the evaluation of a mutant version of the heat labile *E. coli* enterotoxin (LT, closely related to CT in sequence and mechanism of action), which possesses adjuvanticity but lacks toxicity [32]. The humoral responses detected in saliva and feces, although modest, highlight the potential to protect the mucosal compartments by oral immunization. Further research will optimize and better characterize the humoral response in the mucosal compartments. Additional immunization schemes are ongoing to determine the humoral response reached not only in the gut and saliva but also in the lungs as this is the primary tissue to be protected upon COVID-19 infection. These new schemes are also designed to study the effect of combining parental priming with oral boosting (using a mucosal adjuvant acceptable for human use).

In the vaccinology field, emerging platforms based on photosynthetic organisms are acquiring relevance since these are considered attractive hosts for the synthesis and delivery of antigens, providing the basis for the development of low-cost and easy-to-administer vaccines [33]. For instance, plants have proved to be a viable alternative platform for vaccine production, with the approval of the vaccine against SARS-CoV-2 developed by Medicago Inc. as a key precedent for the field [34]. As for the case of algae-based platforms, this technology has advanced to a lesser extent, but if the efforts to explore this platform increase, the introduction of algae-made biopharmaceuticals is a realistic goal to achieve. Oral vaccines could be formulated with freeze-dried algae or plant material expressing the antigen of interest. This could be especially convenient for booster vaccines used in developing countries, in which cold chain distribution and availability of sterile needles might compromise the coverage and rapid advances for the vaccination campaigns, although an expansion in the research to optimize such an approach is still needed [35].

Interestingly, the material used for the current immunological evaluation was highly stable as neither a significant decrease in the antigen content nor protein degradation occurred as revealed by the Western blot analysis of freeze-dried algae biomass stored for a long period at room temperature; this is in line with other reports indicating that photosynthetic cells may act as stable biocapsules and delivery vehicles for antigens. Therefore, the freeze-dried algae biomass containing the expressed RBD could be used as the basis to produce thermally stable vaccines, an attribute that will highly decrease the vaccine cost. The use of gelatin capsules containing freeze-dried biomass for oral immunization has been assessed with promising results. This approach will be highly suitable for the design of oral boosting formulations [36].

Our algae-made RBD is a candidate that merits further evaluations to assess if it could serve as an oral boosting agent in parenterally vaccinated mice such that it could be proposed as a booster agent for the population already vaccinated but requiring boosting to sustain immunity. Therefore, a key perspective consists in the evaluation of the algae-made RBD as a booster in test animals vaccinated with RNA- or Adenoviral-vaccines, as these are the most currently used vaccines to fight the current pandemic. Ongoing efforts are focused on evaluating the neutralizing potential of the antibodies induced by the algae-made RBD by using a pseudovirus-based assay.

RBD has been expressed in *Chlamydomonas* by an approach consisting of nuclear-stable transformation, in which a linear vector is randomly integrated in the genome to code a fusion protein called RBD::mClover, comprising of RBD and a fluorescent protein for easy monitorization of expression [37]. For this purpose, a double selection of the transformed clones was performed using hygromycin and zeocin. Constructs aimed at inducing different subcellular localizations (chloroplast and ER-retention) and secretion of the protein were assessed, and the ER-retention approach was deemed as the better approach, allowing the expression of RBD capable of binding the ACE2 receptor and achieving yields up to 31 μg/g of fresh algae biomass, while in partially purified extracts the yield achieved was 1.8 μg/g of fresh algae biomass.

Another possibility to explore consists of chloroplast transformation, which relies on site-directed integration in the plastome, which is present in hundreds of copies, and thus, high expression is favored by this attribute. Although such an approach offers attractive yields, it requires extensive characterization of many clones to select the most stable and productive lines [38].

In contrast, our approach relies on transient expression, which implies a short production time and no need for clone selection and characterization or fusing the antigen to a protein partner, but still has competitive yields (up to 4 µg/g). RBD has also been transiently expressed in *N. benthamiana* with yields of up to 8 μg of pure antigen/g leaf mass [39].

In conclusion, we proved the concept of using RBD expressed in algae to induce antibodies able to recognize the S protein. To the best of our knowledge, this is the first report on the immunogenicity assessment for an algae-made antigen against SARS-CoV-2. The positive outcomes from this study justify further evaluations, for instance, assessing the potential of this vaccine to act as a booster agent in test mice primed with the vaccines approved for emergency use.

## 4. Materials and Methods

### 4.1. Algae Transformation

*Chlamydomonas reinhardtii* was grown in sterile Tris-acetate phosphate (TAP) medium (PhytoTech Labs, Lenexa, KS, USA) and incubated at 25 ± 2 °C in an artificial climate shaking incubator provided with cool-white, fluorescent light of 80–100 µmol photons/m^2^∙s with a photoperiod of 16 h/8 h (light/darkness). The culture was monitored and allowed to grow for 4 to 6 days until the cell density attained the log phase (OD at 680 nm of 0.7–0.8). The log phase culture was further used for scale-up in 2 × 1 L conical flasks with an occupancy of 500 mL of sterile TAP culture medium in each flask. Each flask was inoculated with 5% (*v*/*v*) of log phase *C. reinhardtii* and allowed to grow in the above-mentioned conditions until an OD 680 nm = 0.8 was reached. The log phase cells of *C. reinhardtii* were further used for transient expression of SARS-CoV-2 RBD by *Agrobacterium*-mediated gene transformation.

### 4.2. Transient Transformation in Chlamydomonas reinhardtii

*Agrobacterium*-mediated transformation was carried out using the geminiviral plant expression vector pBYR2e carrying the SARS-CoV-2 RBD gene [28,39], which was kindly provided by Dr. Waranyoo Phoolcharoen, Faculty of Pharmaceutical Sciences, Chulalongkorn University, Thailand. The *A. tumefaciens* strain carrying the pBYR2E:SARS-CoV-2-RBD vector was grown in LB media with kanamycin (50 mg/L) and rifampicin (50 mg/L) at 28 °C in a dark rotatory shaker at 150 rpm until an OD 600 nm of 1.0 was attained. In each flask of log phase algal culture, 5 mL of *Agrobacterium* culture with an OD 600 nm = 1.0 was added and allowed to grow for 24 h on a rotatory shaker in the presence of 100 µM acetosyringone for effective transformation. The antibiotic cefotaxime (250 mg/L) was added 24 h post-infection, and the culture was allowed to grow until harvested after 24 h of antibiotic addition.

### 4.3. RBD Detection and Stability Assessment

The biomass from transformed *C. reinhardtii* was harvested by centrifugation at 5000 rpm for 10 min at 4 °C and subsequently washed with sterile ice-cold water 3 to 5 times to remove the traces of antibiotic and bacterial cells. The transformed biomass was subjected to protein extraction, SDS-PAGE, confirmation by Western blot, and ELISA of soluble protein extracts for the quantification of RBD content using an anti-RBD-specific serum, following previously described protocols [28]. The biomass transformed was stored at −80 °C for 2 days and then freeze-dried overnight in a LABCONCO device at 0.002 mBar and 4 °C for the collector temperature.

The biomass from the freeze-dried *C. reinhardtii* expressing RBD was stored at room temperature for 15 months and subsequently subjected to protein extraction and Western blot assay to evaluate the integrity of RBD following the protocols reported by Malla et al. [28].

### 4.4. Immunization Schemes

The algae-made RBD was evaluated in 12-week-old female BALB/c mice, which were maintained according to the national regulations for animal care (SAGARPA, NOM-062-ZOO-1999, Mexico). The immunization protocol was approved by the Research and Teaching Committee of the Chemical Faculty at the University of San Luis Potosí, Mexico (Register Number: CEID-2020-07R1). The experimental groups were randomly established using five mice per group. Four doses were administered biweekly by subcutaneous (s.c.) or oral (p.o.) routes on days 1, 14, 28, and 42. Groups C. RBD s.c. and C. RBD + Al(OH)_3_ s.c. received in the back total protein extracts (200 μL), which were obtained by resuspending in PBS 10 mg of freeze-dried *C. reinhardtii* expressing RBD (containing 0.2 μg of RBD), followed by sonication (15 cycles comprising of 10 s pulses with 10 s off intervals at a 25% amplitude), and clarification by centrifugation at 13,000× *g* for 5 min. The C. RBD p.o. and C. RBD + CT p.o. groups received intragastrically 10 mg of freeze-dried C. RBD (containing 0.2 μg of RBD) resuspended by vortexing in PBS. In the latter, cholera toxin (CT) was used as adjuvant (1 μg). The PBS group received the vehicle alone (PBS). Test animals were sacrificed on day 56, except for the groups PBS and C. RBD + CT p.o., which were sacrificed on day 200 to assess the long-lasting immunity (Table 1).

### 4.5. Serum, Saliva, and Feces Sample Preparation

Blood samples were withdrawn from all mice groups on days 41 and 56 by punction in the tail. After clot formation, samples were centrifuged at 3500× *g* for 10 min, and the sera obtained were stored at −20 °C until antibody determination. Additionally, for the C. RBD + CT p.o. and PBS groups, feces and saliva samples were taken on day 56 as follows: Feces extracts were obtained through the collection of 100 mg of feces from immunized mice, which were resuspended in 500 μL of ice-cold PBS supplemented with 5% fat-free dry milk and 1 mM of PMSF. The feces were dispersed using a plastic device, and the suspension was centrifuged at 7500× *g* and 4 °C for 15 min for subsequent separation of the supernatant, which was used to run ELISA. Saliva samples were obtained from mice anesthetized with isoflurane; the mice were then subjected to mouth wash with 120 μL of PBS. The washes obtained were added with 1 mM PMSF. Feces and saliva samples were immediately plated for antibody determination by ELISA. Some test animals (*n* = 5) were sacrificed on day 200 to determine long-lasting humoral responses in sera as described above.

### 4.6. Antibody Measurement

Antibody levels were determined by indirect ELISA to detect IgG and/or IgA against the S461-493 peptide and Spike protein (Sinobiological. Cat. No. 40589-V08H4, 40589-V08B16, and 40591-V08H41 for Wuhan, Delta, and Omicron, respectively) in serum, saliva, and feces. For this purpose, 96-well polystyrene plates were coated overnight at 4 °C with 100 ng/well of the S461-493 peptide hereafter called P6, or spike (Delta, Omicron, or Wuhan) protein in carbonate buffer (15 mM Na_2_CO_3_, 35 mM NaHCO_3_, pH 9.6). Before the following steps, plates were washed three times with PBS + Tween 0.05% (PBS-T). Plates were then blocked with 5% fat-free dry milk in PBS for 2 h at room temperature. Serial dilutions of sera (1:50–1:200) or saliva and fecal extracts (1:2–1:8) in PBS were added and incubated at 4 °C overnight. Afterward, for all samples, secondary antibodies detected using a goat horseradish peroxidase-conjugated anti-mouse IgG or IgA diluted in PBS (1:2000) were used. Additionally, for sera samples, goat horseradish peroxidase-conjugated anti-mouse IgG1 or IgG2a was used for secondary labeling (dilution 1:2000). Finally, an ABTS substrate solution [0.6 mM 2,20-azino-bis(3-ethylbenzothiazoline-6-sulfonic acid) + 0.1 M citric acid + 1 mM H_2_O_2_, pH 4.35] was added and after an incubation for 30 min at room temperature, OD values at 405 nm were measured using MultiskanR FC equipment (Thermo Scientific, Waltham, MA, USA). Statistical differences were determined using one-way ANOVA employing the GraphPad Instat version 3.1; Dotmatics: Boston, MA, USA, 2009 (*p* < 0.05).

## Figures and Tables

**Table 1:** Description of experimental groups. Mice were randomly assigned to the treatments described below.

Group	Route	RBD Dose	Adjuvant	Vehicle PBS
C. RBD s.c.	s.c.	0.2 µg *	-	✓
C. RBD Al(OH)_3_ s.c.	s.c.	0.2 µg *	Al(OH)_3_ 1:5 (*v*/*v*)	✓
C. RBD p.o.	p.o.	0.2 µg ^+^	-	✓
C. RBD CT p.o.	p.o.	0.2 µg ^+^	CT 1:1 (*v*/*v*)	✓
PBS	s.c.	-	-	✓

**Abbreviations:** C. RBD: *Chlamydomonas*-made RBD given in the form of algae total soluble protein extract; s.c.: subcutaneous; p.o.: per os; DW: Dried Weight; CT: Cholera toxin. * as soluble antigen. ^+^ encapsulated in algae cells.

## Data Availability

Data is contained within the article.

## References

[1] World Health Organization WHO Coronavirus (COVID-19) Dashboard. https://covid19.who.int.

[2] Pollard A.J., Bijker E.M. (2021). A guide to vaccinology: From basic principles to new developments. Nat. Rev. Immunol..

[3] Tregoning J.S., Flight K.E., Higham S.L., Wang Z., Pierce B.F. (2021). Progress of the COVID-19 vaccine effort: Viruses, vaccines and variants versus efficacy, effectiveness and escape. Nat. Rev. Immunol..

[4] Sharif N., Alzahrani K.J., Ahmed S.N., Dey S.K. (2021). Efficacy, immunogenicity and safety of COVID-19 vaccines: A systematic review and meta-analysis. Front. Immunol..

[5] Kashte S., Gulbake A., El-Amin Iii S.F., Gupta A. (2021). COVID-19 vaccines: Rapid development, implications, challenges and future prospects. Hum. Cell.

[6] Koirala A., Joo Y.J., Khatami A., Chiu C., Britton P.N. (2020). Vaccines for COVID-19: The current state of play. Paediatr. Respir. Rev..

[7] Ibarrondo F.J., Hofmann C., Fulcher J.A., Goodman-Meza D., Mu W., Hausner M.A., Ali A., Balamurugan A., Taus E., Elliott J. (2021). Primary, recall, and decay kinetics of SARS-CoV-2 vaccine antibody responses. ACS Nano.

[8] Zhang C., Maruggi G., Shan H., Li J. (2019). Advances in mRNA vaccines for infectious diseases. Front. Immunol..

[9] Soleimanpour S., Yaghoubi A. (2021). COVID-19 vaccine: Where are we now and where should we go?. Expert Rev. Vaccines.

[10] Alturaiki W. (2022). Considerations for novel COVID-19 mucosal vaccine development. Vaccines.

[11] Karczmarzyk K., Kęsik-Brodacka M. (2022). Attacking the intruder at the gate: Prospects of mucosal anti SARS-CoV-2 vaccines. Pathogens.

[12] van Doremalen N., Purushotham J.N., Schulz J.E., Holbrook M.G., Bushmaker T., Carmody A., Port J.R., Yinda C.K., Okumura A., Saturday G. (2021). Intranasal ChAdOx1 nCoV-19/AZD1222 vaccination reduces viral shedding after SARS-CoV-2 D614G challenge in preclinical models. Sci. Transl. Med..

[13] An D., Li K., Rowe D.K., Diaz M., Griffin E.F., Beavis A.C., Johnson S.K., Padykula I., Jones C.A., Briggs K. (2021). Protection of K18-hACE2 mice and ferrets against SARS-CoV-2 challenge by a single-dose mucosal immunization with a parainfluenza virus 5-based COVID-19 vaccine. Sci. Adv..

[14] Park J.G., Oladunni F.S., Rohaim M.A., Whittingham-Dowd J., Tollitt J., Hodges M., Fathallah N., Assas M.B., Alhazmi W., Almilaibary A. (2021). Immunogenicity and protective efficacy of an intranasal live-attenuated vaccine against SARS-CoV-2. iScience.

[15] Kumar U.S., Afjei R., Ferrara K., Massoud T.F., Paulmurugan R. (2021). Gold-Nanostar-Chitosan-Mediated Delivery of SARS-CoV-2 DNA vaccine for respiratory mucosal immunization: Development and Proof-of-Principle. ACS Nano.

[16] Jearanaiwitayakul T., Seesen M., Chawengkirttikul R., Limthongkul J., Apichirapokey S., Sapsutthipas S., Phumiamorn S., Sunintaboon P., Ubol S. (2021). Intranasal administration of RBD nanoparticles confers induction of mucosal and systemic immunity against SARS-CoV-2. Vaccines.

[17] Sung J.C., Liu Y., Wu K.C., Choi M.C., Ma C.H., Lin J., He E., Leung D.Y., Sze E.T., Hamied Y.K. (2021). Expression of SARS-CoV-2 Spike protein receptor binding domain on recombinant *B. subtilis* on spore surface: A potential COVID-19 oral vaccine candidate. Vaccines.

[18] Peng K.W., Carey T., Lech P., Vandergaast R., Muñoz-Alía M.Á., Packiriswamy N., Gnanadurai C., Krotova K., Tesfay M., Ziegler C. (2022). Boosting of SARS-CoV-2 immunity in nonhuman primates using an oral rhabdoviral vaccine. Vaccine.

[19] Johnson S., Martinez C.I., Tedjakusuma S.N., Peinovich N., Dora E.G., Birch S.M., Kajon A.E., Werts A.D., Tucker S.N. (2022). Oral vaccination protects against severe acute respiratory syndrome coronavirus 2 in a Syrian hamster challenge model. J. Infect. Dis..

[20] Sui Y., Li J., Zhang R., Prabhu S.K., Andersen H., Venzon D., Cook A., Brown R., Teow E., Velasco J. (2021). Protection against SARS-CoV-2 infection by a mucosal vaccine in rhesus macaques. JCI Insight.

[21] Pitcovski J., Gruzdev N., Abzach A., Katz C., Ben-Adiva R., Brand-Shwartz M., Yadid I., Ratzon-Ashkenazi E., Emquies K., Israeli H. (2022). Oral subunit SARS-CoV-2 vaccine induces systemic neutralizing IgG, IgA and cellular immune responses and can boost neutralizing antibody responses primed by an injected vaccine. Vaccine.

[22] Rosales-Mendoza S., García-Silva I., González-Ortega O., Sandoval-Vargas J.M., Malla A., Vimolmangkang S. (2020). The potential of algal biotechnology to produce antiviral compounds and biopharmaceuticals. Molecules.

[23] Garduño-González K.A., Peña-Benavides S.A., Araújo R.G., Castillo-Zacarías C., Melchor-Martínez E.M., Oyervides-Muñoz M.A., Sosa-Hernández J.E., Purton S., Iqbal H.M.N., Parra-Saldívar R. (2022). Current challenges for modern vaccines and perspectives for novel treatment alternatives. J. Drug. Deliv. Sci. Technol..

[24] Specht E.A., Mayfield S.P. (2014). Algae-based oral recombinant vaccines. Front. Microbiol..

[25] Bañuelos-Hernández B., Monreal-Escalante E., González-Ortega O., Angulo C., Rosales-Mendoza S. (2017). Algevir: An expression system for microalgae based on viral vectors. Front. Microbiol..

[26] Bolaños-Martínez O.C., Mahendran G., Rosales-Mendoza S., Vimolmangkang S. (2022). Current Status and Perspective on the Use of Viral-Based Vectors in Eukaryotic Microalgae. Mar. Drugs.

[27] Diamos A.G., Hunter J.G.L., Pardhe M.D., Rosenthal S.H., Sun H., Foster B.C., DiPalma M.P., Chen Q., Mason H.S. (2020). High level production of monoclonal antibodies using an optimized plant expression system. Front. Bioeng. Biotechnol..

[28] Malla A., Rosales-Mendoza S., Phoolcharoen W., Vimolmangkang S. (2021). Efficient transient expression of recombinant proteins using DNA viral vectors in freshwater microalgal species. Front. Plant Sci..

[29] Beltrán-López J.I., Romero-Maldonado A., Monreal-Escalante E., Bañuelos-Hernández B., Paz-Maldonado L.M., Rosales-Mendoza S. (2016). *Chlamydomonas reinhardtii* chloroplasts express an orally immunogenic protein targeting the p210 epitope implicated in atherosclerosis immunotherapies. Plant Cell Rep..

[30] Pniewski T., Kapusta J., Bociąg P., Wojciechowicz J., Kostrzak A., Gdula M., Fedorowicz-Strońska O., Wójcik P., Otta H., Samardakiewicz S. (2011). Low-dose oral immunization with lyophilized tissue of herbicide-resistant lettuce expressing hepatitis B surface antigen for prototype plant-derived vaccine tablet formulation. J. Appl. Genet..

[31] Sui Y., Bekele Y., Berzofsky J.A. (2021). Potential SARS-CoV-2 immune correlates of protection in infection and vaccine immunization. Pathogens.

[32] Norton E.B., Lawson L.B., Freytag L.C., Clements J.D. (2011). Characterization of a mutant *Escherichia coli* heat-labile toxin, LT(R192G/L211A), as a safe and effective oral adjuvant. Clin. Vaccine Immunol..

[33] Fausther-Bovendo H., Kobinger G. (2021). Plant-made vaccines and therapeutics. Science.

[34] Pillet S., Arunachalam P.S., Andreani G., Golden N., Fontenot J., Aye P.P., Röltgen K., Lehmicke G., Gobeil P., Dubé C. (2022). Safety, immunogenicity, and protection provided by unadjuvanted and adjuvanted formulations of a recombinant plant-derived virus-like particle vaccine candidate for COVID-19 in nonhuman primates. Cell Mol. Immunol..

[35] Gonzalez-Cruz P., Gill H.S. (2021). Demystifying particle-based oral vaccines. Expert Opin. Drug Deliv..

[36] Daniell H., Kulis M., Herzog R.W. (2019). Plant cell-made protein antigens for induction of Oral tolerance. Biotechnol. Adv..

[37] Berndt A.J., Smalley T.N., Ren B., Simkovsky R., Badary A., Sproles A.E., Fields F.J., Torres-Tiji Y., Heredia V., Mayfield S.P. (2021). Recombinant production of a functional SARS-CoV-2 spike receptor binding domain in the green algae *Chlamydomonas reinhardtii*. PLoS ONE.

[38] Larrea-Alvarez M., Young R., Purton S. (2021). A simple technology for generating marker-free chloroplast transformants of the green alga *Chlamydomonas reinhardtii*. Methods Mol. Biol..

[39] Rattanapisit K., Shanmugaraj B., Manopwisedjaroen S., Purwono P.B., Siriwattananon K., Khorattanakulchai N., Hanittinan O., Boonyayothin W., Thitithanyanont A., Smith D.R. (2020). Rapid production of SARS-CoV-2 receptor binding domain (RBD) and spike specific monoclonal antibody CR3022 in *Nicotiana benthamiana*. Sci. Rep..

